# 

**Published:** 1879-03

**Authors:** 


**EDITORS:**

WILLIAM H. BYFORD, A.M., M.D.,
JAS. NEVINS HYDE, A.M., M.D. FERD. C. HOTZ, M.D.
E. FLETCHER INGALS, M.D.

CHICAGO MEDICAL PRESS ASSOCIATION.

AUG. 2D

1875.

CHICAGO:

*PUBLISHED BY THE CHICAGO MEDICAL PRESS ASSOCIATION.*

188 CLARK STREET.

☞**Read the Notice of this Journal among Advertisements.**

